# Statin use as a moderator on the association between metformin and breast cancer risk in women with type 2 diabetes mellitus

**DOI:** 10.1186/s40170-024-00340-8

**Published:** 2024-04-12

**Authors:** Fan Zhang, Geertruida H. de Bock, Gijs W. Landman, Qingying Zhang, Grigory Sidorenkov

**Affiliations:** 1 Department of Epidemiology, University Medical Center Groningen, University of Groningen, Groningen, The Netherlands; 2 Oncology Research Laboratory, Cancer Hospital of Shantou University Medical College, Shantou, People’s Republic of China; 3 Department of Preventive Medicine, Shantou University Medical College, Shantou, People’s Republic of China; 4 Department of Internal Medicine, Gelre Hospital, Apeldoorn, The Netherlands

**Keywords:** Diabetes, Metformin, Statins, Interaction, Breast cancer

## Abstract

**Introduction:**

Metformin and statins are considered as potential agents for prevention of breast cancer, however, existing evidence does not uniformly substantiate this claim, and the data is scarce concerning their interaction in relation to breast cancer risk. This study aims to investigate whether the effect of metformin on breast cancer incidence varied by statin use among women with type 2 diabetes mellitus (T2DM).

**Methods:**

This study included women with T2DM, without a history of cancers, and followed up for more than one year from the Zwolle Outpatient Diabetes project Integrating Available Care (ZODIAC) for the period 1998–2014. The dataset was structured using a person-time approach, where the cumulative medication usage was annually updated for each person. The extended Cox proportional hazards models were employed, reporting adjusted hazard ratios (HR) with 95% confidence intervals (CI).

**Results:**

During a median follow-up of 5 years, 515 of 29,498 women received a breast cancer diagnosis. Each additional year of metformin or statins use corresponded to a decrease in breast cancer incidence, while the magnitude attenuated over time. Noteworthily, statin use modified the effect of metformin on breast cancer incidence. For instance, after 5 years of follow-up, one-year increase of metformin use among women who used statins for 3 years was linked to a substantially reduced breast cancer risk (HR, 95% CI: 0.88, 0.84–0.93), however, there was no significant decrease in risk for those non-statins users (HR, 95% CI: 0.96, 0.89–1.04).

**Conclusions:**

Extending metformin or statin usage by one year conferred breast cancer protection in women with T2DM. Enhanced protective effect of metformin was observed among those who also use statins. These results suggest the potential of combined metformin and statin therapy as promising breast cancer prevention strategies.

**Supplementary Information:**

The online version contains supplementary material available at 10.1186/s40170-024-00340-8.

## Introduction

Despite improved prognosis, breast cancer remains a challenging disease, as its incidence in women is increasing globally over the last three decades [1]. Particularly in women with type 2 diabetes mellitus (T2DM), the risk of breast cancer is elevated by 14–25% [2–4]. Therefore, preventive strategies on modifiable risk factors related to breast cancer would have substantial benefit.

Metformin, a first-line drug for T2DM treatment, has shown anti-cancer effects ex vivo, but mixed findings yielded in observational studies regarding breast cancer [5–7]. Two previous randomized controlled trials (RCTs), not specially designed for this issue, observed no reduction in breast cancer risk by metformin use [8]; but one recent RCT uncovered that metformin intervention resulted in favourable changes in breast cancer risk factors, such as reduction in waist circumference and non-dense breast volume [9]. Another commonly used medication, statins, tended to be concluded insignificantly associated with breast cancer risk [10], while some negative and positive associations/effects were previously reported in either observational studies [11, 12] or RCT [13]. Thus, the efficacy of metformin and statins in the prevention of breast cancer still remains unclear.

In vitro experiments indicated metformin and statins share common targets and pathways, like AMP-activated protein kinase signalling, to inhibit cell proliferation and trigger apoptosis in cancer cells [14, 15]. One recent paper provided direct evidence that the combined use of metformin and simvastatin exerts a more potent suppression on endothelin 1-induced hypoxia and angiogenesis in breast cancer cell lines compared to each drug alone [16]. Regarding epidemiological evidence, as far as we know, one cohort study conducted in the Finnish nationwide diabetes database, mentioned no interaction effect between these two drugs on breast cancer incidence but without elaborating analysis details [17]. So far, there is a paucity of evidence answering the question: whether the association between metformin and the risk of breast cancer could be modified by statins. This, however, was confirmed in two studies working on men with T2DM considering prostate cancer incidence [18, 19].

Thus, we utilized a real-world dataset and conducted a cohort study to evaluate the associations of metformin and statins use with breast cancer risk, and particularly to examine the interaction and potentially synergistic effects of the combination treatment with metformin and statins on breast cancer risk in women with T2DM.

## Materials and methods

### Study design and setting

A population-based cohort study was performed in women diagnosed with T2DM, who were recruited in the Zwolle Outpatient Diabetes project Integrating Available Care (ZODIAC), an annual benchmarking database for 731 general practitioners (GP) in Dutch primary care, for the period 1998 to 2014. Data were linked to the Netherlands Cancer Registry and the Dutch Personal Record Database for cancer and mortality data. This research required no ethics committee approval (**see Supplementary Material for explanations**).

### Baseline date and follow up

The baseline is defined as July 1th in the year when the clinical data was available in ZODIAC database. Follow-up started at baseline, and ended at the date of breast cancer diagnosis, death or loss-to-follow-up (the year when no more clinical data were present for a patient, namely body mass index (BMI), smoking status, any blood/urine measures, blood pressures, prescriptions of any medication in this cohort till July 1th, 2014), or July 1th, 2015, whichever came first. Censoring occurred when a woman died or did not have follow-up data (no measurements or prescriptions) before a diagnosis of breast cancer at the end of the follow up.

### Patient selection

The following inclusion criteria were applied in this study: (1) women were diagnosed with T2DM at baseline; (2) to ensure that patients had ever visited a general practice within a study period, we only included patients who had at least one of the following records: BMI, smoking status, blood/urine measures, or blood pressures between 1998 and 2014. Patients were excluded if they satisfied at least one of the following criteria (1) they had a history of cancer other than non-melanoma skin cancer at baseline [20]; (2) they had follow up ≤ one year after the baseline; (3) to ensure that medication was recorded, we excluded those patients who never received drug prescriptions in the study period (between 1998 and 2014). Of note, a history of medication usage before baseline is unavailable, and patients included in this cohort may not be new users regarding metformin and statins.

### Data collection

#### Data at baseline

Baseline data were collected with different strategies to limit missingness as follows: age at baseline, BMI and smoking status as values taken nearest to the baseline date, duration of diabetes as a period between the registered date of diabetes and the baseline (the registered date was around or before the baseline), first records within one year around the baseline of glycated hemoglobin A (HbA1c), low-density lipoprotein cholesterol (LDL-C) and estimated glomerular filtration rate (eGFR), and a history of cardiovascular disease, hypertension and cancer before or at the baseline. Certified labs performed the sample analyses for laboratory measurements. LDL-C was routinely calculated using the Friedewald equation [21]. Further details are included in Fig. S1 and the **Supplementary Material**. Baseline values of HbA1c, LDL-C and eGFR were only used for comparisons between women with and without breast cancer.

#### Time-dependent data during the follow up

Time-dependent data on the following variables were collected: use of metformin, statins, sulfonylurea, and insulin, as well as values of HbA1c, LDL-C, and eGFR. These data were treated as time-dependent further in the models.

The dataset was structured in a person-time interval format, with each interval spanning one year. Within each person-time interval, exposure to metformin was defined as yes or no. As the data were collected at annual benchmarks, cumulative exposure at the beginning of each interval was calculated in years as the sum over all the intervals where a prescription was present since the first known prescription at baseline. To avoid a small sample size for the category of patients receiving metformin for a long period, we truncated the cumulative exposure at six years of follow up. That is, patients who received metformin for six or more years were assigned to one category. A similar approach was applied to the other drugs: statins, sulfonylurea and insulin. More details are presented in **Supplementary Material**. Regarding blood measures, values were updated for each interval. In case of a missing value in a certain interval before a patient has been censored or received a diagnosis of breast cancer, the value from the last interval was used instead.

#### Latency period

To appropriately classify metformin and statin exposure with respect to breast cancer diagnosis, a one-year (365 days) latency period was applied. It means that any metformin or statin prescription which was prescribed in the one-year period preceding breast cancer diagnosis, death, or censoring, was not considered as exposure because breast cancer pathogenesis is likely have already been initiated during that period, and any exposure during this period was assumed to not influence breast cancer diagnosis. This latency period was also applied when collecting blood measures [22].

### Outcome and exposure

The outcome of interest was time to breast cancer diagnosis. The exposures of interest were metformin, statins and particularly the interaction between metformin and statins.

### Statistical analyses

All data analyses were performed using R version 4.1.0 statistical software. Descriptive statistics were presented as mean (standard deviation, SD) or count (percentage, %). The baseline characteristics of the patients diagnosed with breast cancer were compared with those without diagnosis using independent *t*-tests (continuous variables) or chi-square tests (categorical variables; the group of missing values was not accounted).

Cox proportional hazards (PH) models were conducted to assess the interaction between two time-dependent variables: metformin and statins, alongside their individual association with breast cancer occurrence, presenting hazard ratios (HRs) and 95% confidence intervals (CIs). As these two variables (i.e., metformin and statins) violated the PH assumption, the extended Cox PH models were applied allowing a regression coefficient (log-scale HR) to vary as a flexible function of time. Specifically, the interaction term between metformin/statins and a 3-knot restricted cubic splines function of follow-up time was included into the model, so that time-varying coefficient of metformin and statins were estimated. To evaluate the interaction between metformin and statins, the multiplicative interaction term of metformin × statins was added as a time-independent variable. Additionally, the additive interaction on the same scale was calculated by the relative excess risk due to interaction (RERI) based on model 2 estimates [23, 24]. As there were missing values (missing rate in total, %) in BMI (22.3%), smoking status (1.0%), HbA1c (5.8%), and LDL-C (4.7%) at baseline, we imputed those for the Cox models using multiple imputation by chained equations generating five imputed datasets with the “mice” R-package (see **Supplementary Material**).

Except drugs and age, other covariates were categorized. The following were categorized into three groups using two cutoffs: 7.0 and 8.5% for HbA1c, 2.6 and 3.6 in mmol/L for LDL-C as suggested by the literature [25], 60 and 90 units for eGFR, 2 and 5 years for duration of diabetes at baseline (< 2, 2–5, ≥ 5 years). For these variables with three groups, the lowest level was the reference. The rest, namely BMI (cut-off value: 30 kg/m^2^), smoking status, year at baseline (cutoff value: 2018), a history of cardiovascular disease (yes/no), and a history of hypertension (year/no), served as dichotomous variables in the analyses.

Regarding model fitting, the time-varying associations of metformin or statins with breast cancer risk were estimated by looking at the main effects of the medications use and their interaction with time. The time-varying association was first estimated in a model including metformin (or statins) and time without other covariates (Crude model), then both metformin and statins were included with and without the interaction term between metformin and statins (Model 1), followed by adjustment for other potential confounders, namely age, BMI, smoking status, a history of cardiovascular disease, a history of hypertension, and duration of diabetes, year at baseline, time-dependent measures of HbA1c, LDL-C, eGFR, sulfonylurea and insulin (Model 2). By adding the interaction term between metformin and statins, the time-varying association of metformin was conditional on statins. Proportionality was met.

### Sensitivity analyses

Sensitivity analyses were separately performed in two subsets of cohort: (1) women aged ≥ 55 years old at baseline, since post- and pre-menopausal breast cancer may have distinct etiology; (2) women with a diabetes duration < 2 years at baseline, to reduce the possible bias caused by a missing history of metformin. Another sensitivity analysis was conducted when considering inverse probability weights for balancing confounders across metformin (details in **Supplementary Material**).

## Results

### Patient characteristics at baseline

**Fig. 1:**
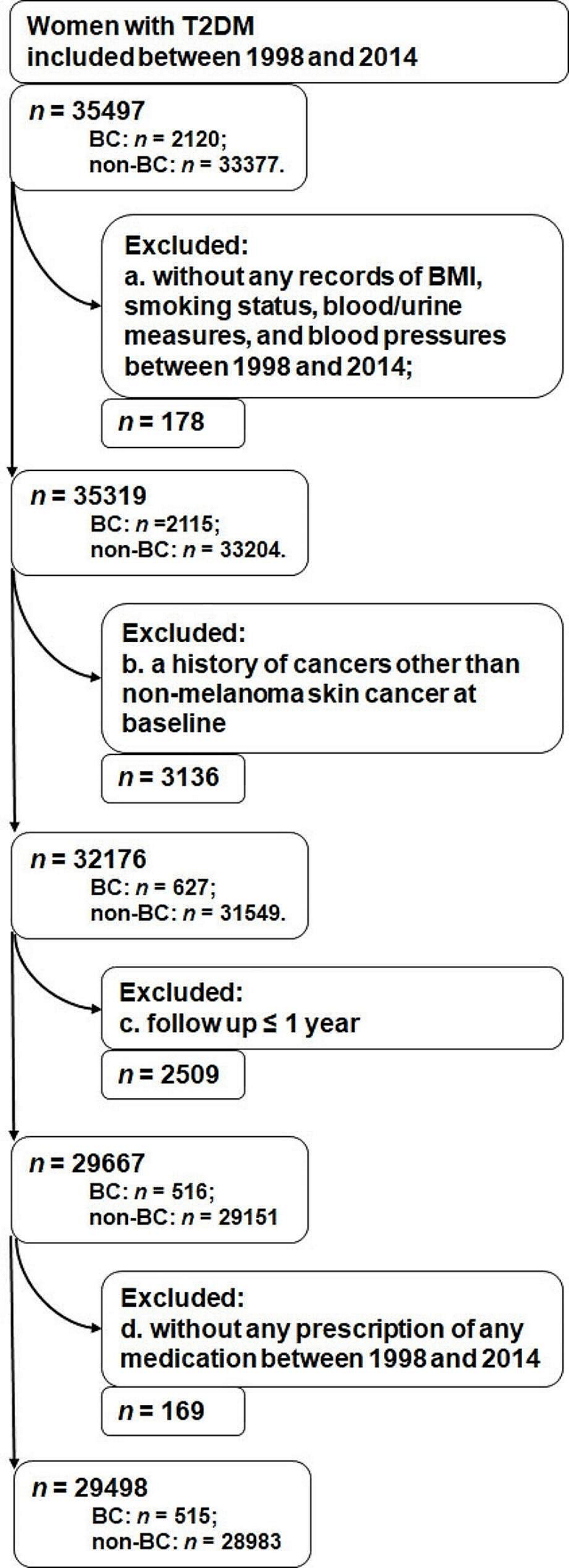
Flow chart of patients selection Abbreviations: BC: breast cancer; BMI: body mass index; T2DM: type 2 diabetes mellitus

A total of 35,497 women with T2DM were recruited; of them, women were further excluded if (1) they had no records of any of the following: BMI, smoking status, blood/urine measures, and blood pressures (*n* = 178); (2) they had a history of cancer other than non-melanoma skin cancer at baseline (*n* = 3136); (3) they were followed for no more than one year (*n* = 2509); (4) they never received a prescription of any medication between 1998 and 2014 (*n* = 169). Consequently, 29,498 women with T2DM formed the study cohort (Fig. 1) with a median follow up of 5.0 years (mean: 5.6 years; range: 1.1–17.0 years), and breast cancer was detected in 515 women. Out of the total, 344 women received diagnoses within five years following the baseline.

As shown in Table 1, women diagnosed with breast cancer were more likely to have a duration of diabetes between 2 and 5 years (29.13% vs. 23.46%, *P* = 0.003), be recruited before 2008 (52.62% vs. 33.83%, *P* < 0.001), have a slightly lower HbA1c at baseline (6.64 ± 0.92% vs. 6.77 ± 1.44%, *P* = 0.003), and not receive metformin at baseline (50.87% vs. 44.05%, *P* = 0.002). Regarding continuous duration of diabetes, a shorter duration was seen in women with breast cancer, in contrast to those without breast cancer (3.81 ± 4.37 vs. 4.29 ± 5.15 years, *P* = 0.015).

**Table 1:** Characteristics at baseline between patients diagnosed with and without breast cancer in this cohort of women with T2DM

Characteristics		Patients without breast cancer (*n* = 28,983)		Patients with breast cancer (*n* = 515)		*P* values ^†^
		N (%)	mean (SD)	N (%)	mean (SD)	
Basic characteristics						
**Age continuous (years)**			66.66 (12.30)		67.36 (10.35)	0.129
**Year at baseline**						
< 2008		9806 (33.83)		271 (52.62)		< 0.001
≥ 2008		19,177 (66.17)		244 (47.38)		
**BMI categorical (kg/m**^**2**^)						
<30		11,763 (40.59)		214 (41.55)		0.553
≥ 30		10,733 (37.03)		208 (40.39)		
missing		6487 (22.38)		93 (18.06)		
**BMI continuous (kg/m**^**2**^)			30.52 (6.26)		30.64 (6.04)	0.668
**Smoking status**						
no		23,405 (80.75)		409 (79.42)		0.666
yes		5305 (18.30)		98 (19.03)		
missing		273 (0.94)		8 (1.55)		
**A history of cardiovascular diseases**						
no		23,596 (81.41)		418 (81.17)		0.931
yes		5387 (18.59)		97 (18.83)		
**A history of hypertension**						
no		16,696 (57.61)		315 (61.17)		0.115
yes		12,287 (42.39)		200 (38.83)		
**Duration of diabetes categorical (years)**						
< 2		12,614 (43.52)		224 (43.50)		0.003
≥ 2, <5		6798 (23.46)		150 (29.13)		
≥ 5		9571 (33.02)		141 (27.38)		
**Duration of diabetes continuous (years)**			4.29 (5.15)		3.81 (4.37)	0.015
**HbA1c categorical (%)** ^**‡**^						
< 7.0		18,969 (65.45)		364 (70.68)		0.061
≥ 7.0, < 8.0		6873 (23.71)		101 (19.61)		
≥ 8.0		1463 (5.05)		27 (5.24)		
missing		1678 (5.79)		23 (4.47)		
**HbA1c continuous (%)** ^**‡**^			6.77 (1.44)		6.64 (0.92)	0.003
**LDL-C categorical (mmol/L)** ^**‡**^						
< 2.6		12,760 (44.03)		224 (43.50)		0.861
≥ 2.6, < 3.6		9343 (32.24)		155 (30.10)		
≥ 3.6		5527 (19.07)		96 (18.64)		
missing		1353 (4.67)		40 (7.77)		
**LDL-C continuous (mmol/L)** ^**‡**^			2.76 (1.60)		2.77 (0.98)	0.876
**eGFR (mL/min/1.73m**^**2**^)^**‡**^						
< 60		6675 (23.03)		128 (24.85)		0.347
≥ 60, < 90		14,292 (49.31)		262 (50.87)		
≥ 90		7466 (25.76)		115 (22.33)		
missing		550 (1.90)		10 (1.94)		
**Metformin** ^**‡**^						
no		12,767 (44.05)		262 (50.87)		0.002
yes		16,216 (55.95)		253 (49.13)		
**Sulfonylurea** ^**‡**^						
no		20,428 (70.48)		359 (69.71)		0.739
yes		8555 (29.52)		156 (30.29)		
**Insulin** ^**‡**^						
no		26,053 (89.89)		474 (92.04)		0.126
yes		2930 (10.11)		41 (7.96)		
**Statins** ^**‡**^						
no		13,552 (46.76)		249 (48.35)		0.501
yes		15,431 (53.24)		266 (51.65)		
**Tumor Characteristics**						
**TNM stage**						
0				50 (9.71)		
1				188 (36.50)		
2				182 (35.34)		
3				62 (12.04)		
4				25 (4.85)		
unclear				8 (1.55)		
**ER**						
negative				60 (11.65)		
positive				382 (74.17)		
unclear				73 (14.17)		
**PR**						
negative				102 (19.81)		
positive				289 (56.12)		
unclear				124 (24.08)		
**HER2**						
negative				373 (72.43)		
positive				48 (9.32)		
unclear				94 (18.25)		

^†^*P* values were from *t* test for continuous variables and chi-square test for categorical variables. ^‡^ Blood measures (namely HbA1c, LDL-C and eGFR) and medication use (yes/no) (namely metformin, sulfonylurea, insulin and statins) in the first time interval were used as baseline values here

Abbreviations: BMI: body mass index; eGFR: estimated glomerular filtration rate; ER: estrogen receptor; HbA1c, glycated hemoglobin A; HER2: human epidermal growth factor receptor-2; LDL-C, low-density lipoprotein cholesterol; PR: progesterone receptor; SD: standard deviation; TNM: tumor, node, metastasis

### Time-varying association of metformin or statins use with breast cancer risk

Before considering the interaction term between metformin and statins, both the crude model and the multivariate models obtained similar estimates on metformin’s and statins’ time-varying associations with breast cancer risk (Table S1 and Fig. S2). That is, one more year of metformin/statins prescription was associated with a lower risk of breast cancer, but the magnitude decreased over time. As for the main effect of metformin, HRs were 0.82 (95% CI: 0.75–0.90) after 3 years of follow-up, and 0.88 (95% CI: 0.83–0.93) after 5 years of follow-up (Fig. S2A and Table 2). As for the main effect of statins, HRs were 0.85 (95% CI: 0.77–0.93) after 3 years of follow-up, and 0.95 (95% CI: 0.89–1.01) after 5 years of follow-up (Fig. S2B and Table S3). HRs for breast cancer risk were further illustrated in the interaction surface plot when no use of metformin or statins served as the reference (Figs. S2C, S2D). Sensitivity analyses yielded similar findings (Table S2 and Table S4).

**Table 2:** Modification of the time-varying association between metformin and breast cancer risk by statins separately after 3-, 5-, 7- and 10-years of follow up

Follow up		Crude model ^†^			Model 1 ^‡^			Model 2 ^§^	
		HR (95% CI)	*P*		HR (95% CI)	*P*		HR (95% CI)	*P*
**3-year**									
**without interaction with statins with interaction with statins**		0.81 [0.74, 0.88]	< 0.001		0.83 [0.76, 0.90]	< 0.001		0.82 [0.75, 0.90]	< 0.001
0		-	-		0.87 [0.79, 0.95]	0.002		0.86 [0.78, 0.94]	0.001
1		-	-		0.84 [0.77, 0.92]	< 0.001		0.84 [0.76, 0.91]	< 0.001
3		-	-		0.79 [0.73, 0.87]	< 0.001		0.79 [0.72, 0.86]	< 0.001
5		-	-		0.75 [0.68, 0.83]	< 0.001		0.74 [0.67, 0.82]	< 0.001
**5-year**									
** without interaction with statins with interaction with statins** ^**¶**^		0.87 [0.83, 0.92]	< 0.001		0.88 [0.83, 0.93]	< 0.001		0.88 [0.83, 0.93]	< 0.001
0		-	-		0.97 [0.90, 1.04]	0.356		0.96 [0.89, 1.04]	0.347
1		-	-		0.94 [0.88, 1.00]	0.047		0.94 [0.88, 1.00]	0.050
3		-	-		0.88 [0.84, 0.93]	< 0.001		0.88 [0.84, 0.93]	< 0.001
5		-	-		0.83 [0.78, 0.89]	< 0.001		0.83 [0.78, 0.89]	< 0.001
**7-year**									
** without interaction with statins with interaction with statins** ^**¶**^		0.90 [0.85, 0.95]	< 0.001		0.90 [0.85, 0.96]	0.001		0.91 [0.86, 0.97]	0.002
0		-	-		1.01 [0.92, 1.10]	0.900		1.01 [0.93, 1.10]	0.820
1		-	-		0.98 [0.91, 1.05]	0.517		0.98 [0.91, 1.06]	0.617
3		-	-		0.92 [0.87, 0.98]	0.005		0.93 [0.87, 0.98]	0.011
5		-	-		0.87 [0.81, 0.92]	< 0.001		0.87 [0.82, 0.93]	< 0.001
**10-year**									
** without interaction with statins with interaction with statins**		0.95 [0.86, 1.05]	0.325		0.95 [0.85, 1.05]	0.283		0.96 [0.87, 1.06]	0.437
0		-	-		1.06 [0.94, 1.20]	0.329		1.08 [0.95, 1.22]	0.245
1		-	-		1.03 [0.92, 1.16]	0.588		1.05 [0.93, 1.17]	0.445
3		-	-		0.97 [0.88, 1.08]	0.588		0.99 [0.89, 1.09]	0.800
5		-	-		0.92 [0.83, 1.01]	0.086		0.93 [0.84, 1.03]	0.163

^†^ Crude model: include cumulative exposure to metformin and its interaction with time; ^‡^ Model 1: include cumulative exposure to metformin and statins, and their interaction with time, with and without the interaction term between metformin and statins; ^§^ Model 2: additionally adjust for baseline information, i.e., age, BMI, smoking status, duration of diabetes, a history of cardiovascular diseases, a history of hypertension, calendar year, as well as updated values of HbA1c, LDL-C, and eGFR, and cumulative exposure to sulfonylurea and insulin to Model 1. ^¶^ Without interaction refers to without including the interaction term between metformin and statins into the model

Abbreviations: CI, confidence interval; HR, hazard ratio

### Statins as a moderator of the association between metformin and breast cancer risk

**Fig. 2:**
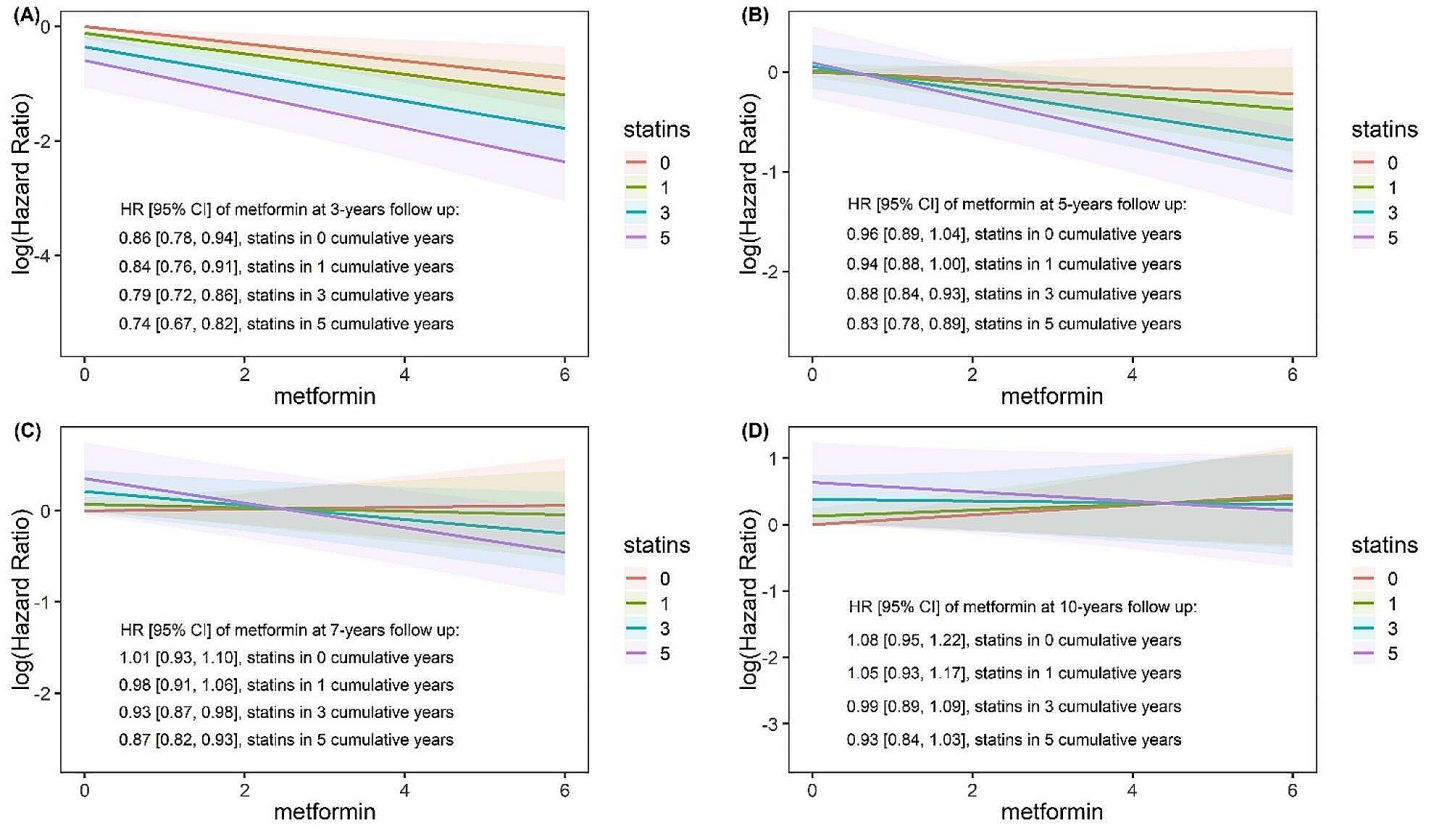
Time-varying associations of metformin and the risk of breast cancer modified by statins In term of the violation of PH assumption, time-varying association between metformin and the risk of breast cancer was evaluated and illustrated separately after 3 (**A**), 5 (**B**), 7 (**C**) and 10 years (**D**) of follow up. In each of them, the y-axis shows the linear predictor of the hazard function for varying values of metformin, holding statins fixed at specific cumulative years, i.e., 0 (red line), 1 (green line), 3 (blue line), 5 years (purple line). The x-axis shows the cumulative usage in years of metformin Abbreviations: CI: confidence interval; HR: hazard ratio

By adding the interaction between metformin and statins, we observed that the association between metformin use and breast cancer risk was modified by statins (HR, 95% CI of the interaction term: 0.97, 0.96–0.99 in model 2, Table S1). This was confirmed by assessing their addictive interaction based on Model 2 in terms that 95% CI of RERI did not cover zero (Table 3). Since the association between metformin and breast cancer was time-varying and statins-dependent, it is illustrated by separately assuming statins use for 0, 1, 3, 5 years after the follow up of 3, 5, 7, and 10 years (Table 2 and Fig. 2). As observed, there was a negative effect of statins on the association between metformin and breast cancer. Particularly, when there is a negative relation between metformin and breast cancer, the slope (HR in log-scale) of metformin was steeper in those with concomitant longer exposure to statins (Fig. 2A, B and C). This implies a stronger magnitude of this main effect of metformin on breast cancer incidence along with an increase in years of statins use. For example (Fig. 2B), after 5 years of follow up, one more year of metformin use was associated with a decrease in breast cancer risk in women with statins use for 5 years (HR: 0.83, 95% CI: 0.78–0.89) and 3 years (HR: 0.88, 95% CI: 0.84–0.93), but that was not seen in those with one-year statins use (HR: 0. 94, 95% CI: 0.88–1.00) or without statins use (HR: 0.96, 95% CI: 0.89–1.04). As for sensitivity analyses, we observed a significant interaction in women aged ≥ 55 years old (HR, 95% CI of the interaction term: 0.97, 0.95–0.99), but not in women with a duration of diabetes < 2 years (HR, 95% CI of the interaction term: 0.98, 0.95-1.00, Table S2). The utilization of inverse probability weights did not result in noticeable differences (Table S4).

**Table 3:** Additive interaction between metformin and statins in cumulative years based on Model 2

Reference level of metformin (years)	Reference level of statins (years)	increment in metformin (years)	increment in statins (years)	RERI	
				RERI	95% CI
1	1	1	1	0.291	0.221, 0.361
3	1	1	2	0.445	0.361, 0.529
5	1	1	3	0.516	0.430, 0.601
1	3	2	1	0.435	0.349, 0.521
3	3	2	2	0.661	0.572, 0.751
5	3	2	3	0.765	0.686, 0.843
1	5	3	1	0.497	0.408, 0.586
3	5	3	2	0.754	0.671, 0.837
5	5	3	3	0.870	0.810, 0.930

RERI: relative excess risk due to interaction; that measures additive interaction between continuous risk factors depending both on their background (reference level) and on the “elevated” levels of exposure (increment in years)

The estimate of the multiplicative interaction between metformin and statins was 0.97 [0.95, 0.99] in model 2, that could be found in Table S1

## Discussion

This study demonstrated that the use of metformin or statins was associated with a lower risk of breast cancer in women with T2DM. However, these estimates diminished over time. Notably, an interaction was detected between metformin and statins that was independent of time. Specifically, the protective effect of metformin on breast cancer risk was more pronounced with prolonged exposure to statins.

### Protective effect of metformin

The association between metformin and breast cancer risk remains a subject of debate, partly due to variations in exposure definition across different studies. Previous studies primarily employed binary analyses (yes/no) of metofrmin use or compared different glucose-lowering medications [8, 26, 27], whereas more recent studies have taken into account the dose and duration of metformin use. Two studies with a nested case-control design both indicated a negative dose-response relationship between metformin and breast cancer risk [28, 29]. However, a study conducted in Israel [7], which employed similar time-dependent techniques as our study, did not support such an association. One potential explanation for this discrepancy is that the Israeli study relied on medication purchase data to determine exposure, while another factor could be that the Israeli study only included patients with incident diabetes and estimated hazard ratios based on the additional mean metformin dose per day.

### Protective effect of statins

In this study only 40% (data not shown) of women who did not achieve LDL-C < 2.6 mmol/L [30], received statins at baseline. Nevertheless, we observed an association between statins and a lower risk of breast cancer. This finding is in contrast to a recent meta-analysis [10]; but it should be noted that several individual studies included in the meta-analysis might introduce biases, such as time-invariant allocation bias [31]. Moreover, previous clinical trials predominantly focused on populations with a high cardiovascular disease risk [32], or their results were inconclusive due to limited sample sizes and short-term use of statins [33, 34]. A recent study indirectly suggested that statin use may be associated with lower mammographic breast density, which serves as a useful biomarker for assessing the effect of chemo-preventive agents on breast cancer risk [35].

### Diminishing magnitude of the protective effect of metformin and statins

The protective effect of metformin and statins use on breast cancer risk diminished over time. This could be attributed to the transient nature of the effect, that is primarily driven by the initiation of treatment with metformin or statins, and the worsening of diabetes over time, that could potentially mask or counteract the benefits of treatment. Of note, due to unavailability of a historical usage, there could be a small fraction of patients who may have been using metformin or statins for years before enrolment. These patients might have a lower risk of breast cancer compared to those new users, that might lead to a potential underestimation of the negative association between metformin/statins and the risk of breast cancer. However, as the estimated negative associations are exceedingly strong, particularly within the first three years of follow-up, we could not exclude the bias possibly due to lower risk of breast cancer among patients who were able to receive and/or adhere to the treatment [36]. Adjustment for accessible confounders was inadequate for fully distinguishing between the direct impact of the treatment and the influence of the patient’s clinical/demographic characteristics linked to an increased likelihood of receiving the treatment. Therefore, while our findings provide further support for the protective effect of metformin and statins on breast cancer risk, it is crucial to interpret this association carefully, taking into consideration disease severity and duration of exposure.

### Interaction between metformin and statins on breast cancer risk

Our study indicated a significant interaction between metformin and statins on a lower breast cancer risk, that was confirmed both in multiplicative and additive scales. However, it is noteworthy that this significance was not observed among women with a diabetes duration of less than 2 years, potentially due to a substantial decrease in a sample size. Nonetheless, a trend indicating the negative association persisted. The synergistic effect of these two drugs is supported by biological experiments that indicated the combination was more efficient than the individual drug on inhibiting tumor cell proliferation, promoting apoptosis, alleviating hypoxia, decreasing angiogenesis, and increasing vessel normalization [16]. However, in an epidemiological perspective, the study conducted in a Finnish cohort of women with T2DM failed to provide supportive finding [17]. Even that, it is important to note that, that study applied distinct study design and exposure definition, and it did not provide details for the interaction analysis Given the limited evidence in this area, further research is warranted to better understand the potential synergistic effects of metformin and statins on breast cancer risk.

### Strengths and limitations

This study was conducted with a relatively large sample size, providing detailed information on medications and laboratory tests that were collected as part of routine GP care in a real-world setting. To address potential biases such as immortal time bias and time-dependent confounding [37, 38], we incorporated time-dependent exposures and covariates in our analysis. Additionally, we took measures to minimize time-lag bias, which commonly arises when comparing metformin with other glucose-lowering drugs due to the order of drug prescriptions during the progression of diabetes [37]. Specifically, we estimated the effect of one additional year of metformin prescription while simultaneously adjusting for the use of sulfonylureas and insulin.

However, it is important to consider several limitations. First, approximately 60% of patients had a diabetes duration exceeding 2 years with no history of using glucose-lowering medications. Part of them could be treatment with lifestyle and diet advice. It is unlikely that this would impact the interaction of metformin and statins since it was assessed after adjusting for the interaction between these medications and time. Second, there was variation in the duration of diabetes between groups, which is known as time-window bias [37]. To address this, we controlled for diabetes duration to mitigate potential confounding from disease severity. Additionally, as our data was organized into one-year intervals, the small difference in duration (approximately half a year on average) is not expected to substantially affect our findings. Third, despite adjusting for an extensive set of confounders, residual confounding from unmeasured factors such as physical activity and dietary factors cannot be entirely excluded. However, it is worth noting that there were no notable differences in the estimates across different adjusted models, indicating that this is unlikely to affect our main findings.

## Conclusions

This study demonstrated a time-varying association of metformin and statins use with the risk of breast cancer in women with T2DM, as well as an interaction between metformin and statins, assesed at both the multiplicative and additive scale. This interaction implies a possible reduction in breast cancer risk for women with T2DM who use both medications simultaneously. These findings provide empirical evidence to support metformin and statins in combination as promising prevention agents for breast cancer.

### Electronic supplementary material

Below is the link to the electronic supplementary material.

Supplementary Material 1

Supplementary Material 2

Supplementary Material 3

## Data Availability

The datasets used and/or analysed during the current study are available from the corresponding author on reasonable request.

## Declarations

### Data accessibility statement

The dataset used in this study is available upon request.

## Abbreviations

BC: breast cancer
BMI: body mass index
CI: confidence interval
eGFR: estimated glomerular filtration rate
ER: estrogen receptor
HR: hazard ratio
HbA1c: glycated hemoglobin A
HER2: human epidermal growth factor receptor-2
LDL-C: low-density lipoprotein cholesterol
PR: progesterone receptor
RCTs: randomized controlled trials
RERI: relative excess risk due to interaction
SD: standard deviation
TNM: tumor, node, metastasis
T2DM: type 2 diabetes mellitus
ZODIAC: Zwolle Outpatient Diabetes project Integrating Available Care

## References

[1] Hu K, Ding P, Wu Y, Tian W, Pan T, Zhang S (2019). Global patterns and trends in the breast cancer incidence and mortality according to sociodemographic indices: an observational study based on the global burden of diseases. BMJ Open.

[2] Larsson SC, Mantzoros CS, Wolk A (2007). Diabetes mellitus and risk of breast cancer: a meta-analysis. Int J Cancer.

[3] Liao S, Li J, Wei W, Wang L, Zhang Y, Li J (2011). Association between diabetes mellitus and breast cancer risk: a meta-analysis of the literature. Asian Pac J Cancer Prev.

[4] Starup-Linde J, Karlstad O, Eriksen SA, Vestergaard P, Bronsveld HK, de Vries F (2013). CARING (CAncer risk and INsulin analoGues): the association of diabetes mellitus and cancer risk with focus on possible determinants - a systematic review and a meta-analysis. Curr Drug Saf.

[5] Col NF, Ochs L, Springmann V, Aragaki AK, Chlebowski RT (2012). Metformin and breast cancer risk: a meta-analysis and critical literature review. Breast Cancer Res Treat.

[6] Tang GH, Satkunam M, Pond GR, Steinberg GR, Blandino G, Schunemann HJ, Muti P (2018). Association of Metformin with breast Cancer incidence and mortality in patients with type II diabetes: a GRADE-Assessed systematic review and Meta-analysis. Cancer Epidemiol Biomarkers Prev.

[7] Dankner R, Agay N, Olmer L, Murad H, Keinan Boker L, Balicer RD, Freedman LS (2019). Metformin Treatment and Cancer Risk: Cox Regression Analysis, with Time-Dependent covariates, of 320,000 persons with Incident Diabetes Mellitus. Am J Epidemiol.

[8] Home PD, Kahn SE, Jones NP, Noronha D, Beck-Nielsen H, Viberti G (2010). Experience of malignancies with oral glucose-lowering drugs in the randomised controlled ADOPT (a diabetes outcome progression trial) and RECORD (Rosiglitazone Evaluated for Cardiovascular outcomes and Regulation of Glycaemia in Diabetes) clinical trials. Diabetologia.

[9] Tapia E, Villa-Guillen DE, Chalasani P, Centuori S, Roe DJ, Guillen-Rodriguez J (2021). A randomized controlled trial of metformin in women with components of metabolic syndrome: intervention feasibility and effects on adiposity and breast density. Breast Cancer Res Treat.

[10] Zhao G, Ji Y, Ye Q, Ye X, Wo G, Chen X (2022). Effect of statins use on risk and prognosis of breast cancer: a meta-analysis. Anticancer Drugs.

[11] Murakami R, Chen C, Lyu SY, Lin CE, Tzeng PC, Wang TF (2016). Lovastatin lowers the risk of breast cancer: a population-based study using logistic regression with a random effects model. Springerplus.

[12] McDougall JA, Malone KE, Daling JR, Cushing-Haugen KL, Porter PL, Li CI (2013). Long-term statin use and risk of ductal and lobular breast cancer among women 55 to 74 years of age. Cancer Epidemiol Biomarkers Prev.

[13] Sacks FM, Pfeffer MA, Moye LA, Rouleau JL, Rutherford JD, Cole TG (1996). The effect of pravastatin on coronary events after myocardial infarction in patients with average cholesterol levels. Cholesterol and recurrent events trial investigators. N Engl J Med.

[14] Aljofan M, Riethmacher D (2019). Anticancer activity of metformin: a systematic review of the literature. Future Sci OA.

[15] Dehnavi S, Kiani A, Sadeghi M, Biregani AF, Banach M, Atkin SL (2021). Target AMPK Statins: Potential Therapeutic Approach Drugs.

[16] Liu J, Wang H, Zhang M, Li Y, Wang R, Chen H (2023). Metformin and simvastatin synergistically suppress endothelin 1-induced hypoxia and angiogenesis in multiple cancer types. Cancer Sci.

[17] Hosio M, Urpilainen E, Marttila M, Hautakoski A, Arffman M, Sund R (2019). Association of antidiabetic medication and statins with breast cancer incidence in women with type 2 diabetes. Breast Cancer Res Treat.

[18] Lehman DM, Lorenzo C, Hernandez J, Wang CP (2012). Statin use as a moderator of metformin effect on risk for prostate cancer among type 2 diabetic patients. Diabetes Care.

[19] Chen-Pin W, Javier H, Lorenzo C, Downs JR, Thompson IM, Pollock B, Lehman D. Statins and Finasteride Use differentially modify the impact of metformin on prostate Cancer incidence in men with type 2 diabetes. Ann Transl Med Epidemiol 2014; 1. PMC4300536 25621309

[20] Park YM, Bookwalter DB, O’Brien KM, Jackson CL, Weinberg CR, Sandler DP (2021). A prospective study of type 2 diabetes, metformin use, and risk of breast cancer. Ann Oncol.

[21] de Vries FM, Voorham J, Hak E, Denig P (2015). Adherence to standard-dose or low-dose statin treatment and low-density lipoprotein cholesterol response in type 2 diabetes patients. Curr Med Res Opin.

[22] Raymakers A, Sin DD, Sadatsafavi M, FitzGerald JM, Marra CA, Lynd LD (2020). Statin use and lung cancer risk in chronic obstructive pulmonary disease patients: a population-based cohort study. Respir Res.

[23] Li R, Chambless L (2007). Test for additive interaction in proportional hazards models. Ann Epidemiol.

[24] Katsoulis M, Bamia C (2014). Additive interaction between continuous risk factors using logistic regression. Epidemiology.

[25] Penson PE, Pirro M, Banach M (2020). LDL-C: lower is better for longer-even at low risk. BMC Med.

[26] Tsilidis KK, Capothanassi D, Allen NE, Rizos EC, Lopez DS, van Veldhoven K (2014). Metformin does not affect cancer risk: a cohort study in the U.K. Clinical Practice Research Datalink analyzed like an intention-to-treat trial. Diabetes Care.

[27] van Staa TP, Patel D, Gallagher AM, de Bruin ML (2012). Glucose-lowering agents and the patterns of risk for cancer: a study with the General Practice Research Database and secondary care data. Diabetologia.

[28] Tseng CH (2014). Metformin may reduce breast cancer risk in Taiwanese women with type 2 diabetes. Breast Cancer Res Treat.

[29] Chikermane SG, Sharma M, Abughosh SM, Aparasu RR, Trivedi MV, Johnson ML (2022). Dose-dependent relation between metformin and the risk of hormone receptor-positive, her2-negative breast cancer among postmenopausal women with type-2 diabetes. Breast Cancer Res Treat.

[30] Authors/Task Force M, Guidelines ESCCP, Societies ESCNC (2019). 2019 ESC/EAS guidelines for the management of dyslipidaemias: lipid modification to reduce cardiovascular risk. Atherosclerosis.

[31] Vinogradova Y, Coupland C, Hippisley-Cox J (2011). Exposure to statins and risk of common cancers: a series of nested case-control studies. BMC Cancer.

[32] Bonovas S, Filioussi K, Tsavaris N, Sitaras NM (2005). Use of statins and breast cancer: a meta-analysis of seven randomized clinical trials and nine observational studies. J Clin Oncol.

[33] Arun BK, Gong Y, Liu D, Litton JK, Gutierrez-Barrera AM, Jack Lee J (2016). Phase I biomarker modulation study of atorvastatin in women at increased risk for breast cancer. Breast Cancer Res Treat.

[34] Vinayak S, Schwartz EJ, Jensen K, Lipson J, Alli E, McPherson L (2013). A clinical trial of lovastatin for modification of biomarkers associated with breast cancer risk. Breast Cancer Res Treat.

[35] Lee Argov EJ, Acheampong T, Terry MB, Rodriguez CB, Agovino M, Wei Y (2020). Independent and joint cross-sectional associations of statin and metformin use with mammographic breast density. Breast Cancer Res.

[36] Guilloteau A, Abrahamowicz M, Boussari O, Jooste V, Aparicio T, Quantin C (2021). Impact of time-varying cumulative bevacizumab exposures on survival: re-analysis of data from randomized clinical trial in patients with metastatic colo-rectal cancer. BMC Med Res Methodol.

[37] Suissa S, Azoulay L (2012). Metformin and the risk of cancer: time-related biases in observational studies. Diabetes Care.

[38] Farmer RE, Ford D, Forbes HJ, Chaturvedi N, Kaplan R, Smeeth L, Bhaskaran K (2017). Metformin and cancer in type 2 diabetes: a systematic review and comprehensive bias evaluation. Int J Epidemiol.

