# Supplementary figures and images for: Statin use as a moderator on the association between metformin and breast cancer risk in women with type 2 diabetes mellitus
Source: Cancer Metab. 2024 Apr 12;12:12. doi: 10.1186/s40170-024-00340-8 (PMC11010330; doi:10.1186/s40170-024-00340-8)

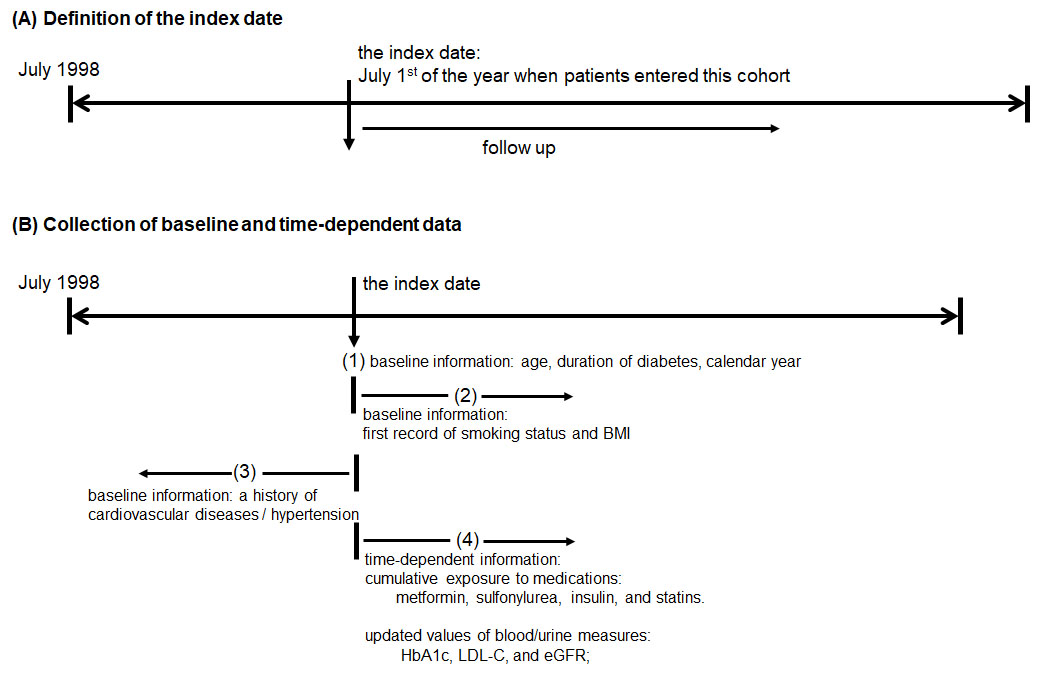

Supplement: Supplementary file 1 — Supplementary Material 1 [file 40170_2024_340_MOESM1_ESM.jpg]

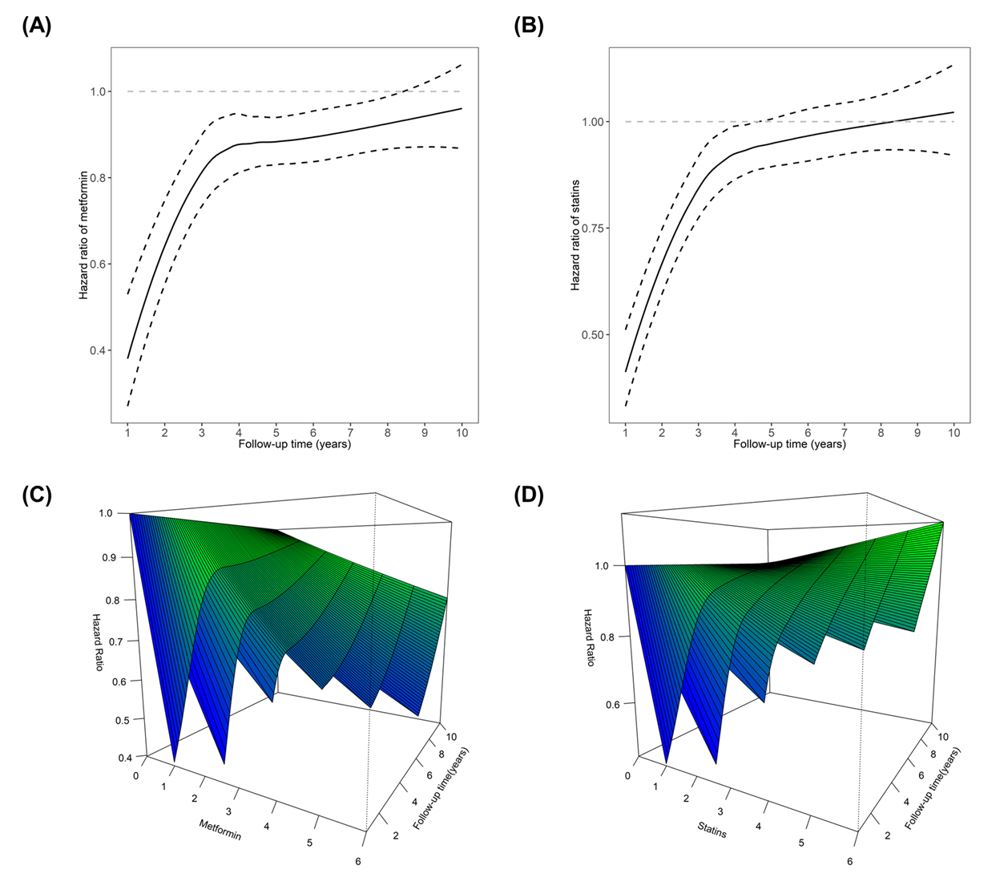

Supplement: Supplementary file 3 — Supplementary Material 3 [file 40170_2024_340_MOESM3_ESM.jpg]
