# Supplementary material for: Statin use as a moderator on the association between metformin and breast cancer risk in women with type 2 diabetes mellitus
Source: Cancer Metab. 2024 Apr 12;12:12. doi: 10.1186/s40170-024-00340-8 (PMC11010330; doi:10.1186/s40170-024-00340-8)
Supplement: Supplementary file 2 — Supplementary Material 2 [file 40170_2024_340_MOESM2_ESM.docx]

**Supplementary Material**

**Methods and Materials:**

**1 Ethics statement**

Patients included in the ZODIAC database consented to the anonymous collection and use of their data for study purposes. The medical ethics committee of Isala evaluated the linkage procedures and exempted this study from formal medical ethics committee review, according to the Dutch Medical Research with Human Subjects Law (*Wet Medisch-wetenschappelijk Onderzoek met mensen*, WMO; METC reference numbers 16.12216 and 16.12214). All procedures were conducted according to the Declaration of Helsinki.

**2 Data collection**

**2.1 Data at baseline**

Different strategies were applied to collect information at baseline to limit missing values (**Figure S1**). Age was determined at baseline; for body mass index (BMI) and smoking status, the valid value taken at the date nearest to the baseline during the follow up was collected. Duration of diabetes is a period between the registered date of diabetes and the baseline (the registered date was around or before the baseline). A history of cardiovascular disease was defined as having a history of any positive record (ICPC code) of myocardial infarction (K75, K76), angina pectoris (K74), Heart failure (K77), Heart value disease NOS (K83), Other heart diseases (K84), stroke / cerebrovascular accident (K90), and transient cerebral ischemia (K89) or ever use of anti-platelet medications (ATC code: B01AC). A history of hypertension was determined by any record of ICPC code K86 and K87. A history of cancer was determined by any cancer-related record of ICD code other than 344 (code for non-melanoma skin cancer). A history of diseases aforementioned was positive when there were positive records before or at the baseline. Data on baseline use of metformin, sulfonylurea, insulin and statins were collected and compared for women with and without breast cancer. Women could receive more than one of these medications in one time interval concurrently.

**2.2 Time-dependent data during the follow up**

When calculating the variable ‘cumulative exposure to metformin’, in case of no prescription (a missing value) for one year between years with present prescriptions, an existing prescription at that year was assumed and added to the cumulative exposure calculation. For example, a woman with three years of follow up got prescription of metformin at the first and third year, then we considered the prescription had also been fulfilled at the second year. Otherwise, when there was more than one year of missing prescriptions, the values were considered as true missing and the cumulative exposure from the previous present time interval was carried forward. For example, when a woman with four years of follow up got metformin prescription at the first and the fourth years, we assumed she probably did not get prescriptions between these two years. In this situation, the cumulative exposure of metformin was 1, 1, 1 and 2 during the follow up. Allowing a patient’s exposure status to vary over the follow-up period can result in more statistical power to detect moderate effects, and minimizes the likelihood of biases, such as immortal time bias [1].

**3 Statistical analyses**

The following variables had missing values (missing rate, %) at baseline in women with and without a diagnosis of breast cancer: BMI (18.06% and 22.38%), HbA1c (4.47% and 5.79%), eGFR (1.94% and 1.90%), LDL-C (7.77% and 4.67%), and smoking status (1.55% and 0.94%). Multiple imputation using a chained equations procedure was performed, and five imputed datasets were generated with the “mice” R-package. Since eGFR was likely missing not at random, the missing values of this variable were not imputed, and instead formed another group.

The standardized the inverse probability weights were estimated in the R-package “ipw”. Metformin was set as the primary exposure of interest, and the confounders were baseline values namely age, year of inclusion, BMI, smoking, a history of cardiovascular diseases, hypertension, duration of diabetes, and also updated values namely sulfonylurea use, insulin use, LDL-C, HbA1c and eGFR. Then, we built a Cox regression of metformin and statins, and with and without their interaction when simultaneously considering weights.

**References:**

1. Suissa S. and Azoulay L. Metformin and the risk of cancer: time-related biases in observational studies. Diabetes Care. 2012; 35: 2665-73.

**Legends to figures**

**Figure S1 Definition of the index date at baseline and Data collection.**

Abbreviations: BMI: body mass index; HbA1c, glycated hemoglobin A; LDL-C, low-density lipoprotein cholesterol; eGFR: estimated glomerular filtration rate.

**Figure S2 Time-varying associations of metformin (or statins) and the risk of breast cancer.**

Time-varying associations between metformin (or statins) and the risk of breast cancer simultaneously adjusting for covariates, namely age, year at baseline, BMI, smoking status, duration of diabetes, a history of cardiovascular diseases, a history of hypertension, cumulative exposure to statins (or metformin), sulfonylurea and insulin, HbA1c, LDL-C, and eGFR (Model 2 without the interaction term between metformin and statins). (**A, B**) Two-dimensional plot illustrates the time-varying association between cumulative exposure to metformin (or statins) and the risk of breast cancer. The y-axis refers to HRs of metformin (or statins) estimated from model 2 (without the interaction term between metformin and statins), and the x-axis refers to the follow up in years. (**C, D**) Three-dimensional surface plot showing the relation between metformin (or statins) and follow-up time on the x- and y-axes, respectively. The z-axis shows the HRs for varying values of metformin (or statins) and follow-up time, illustrating their interacting on the risk of breast cancer. For example, when keeping the other covariates constant, model 2 illustrates that HRs for women who used metformin for 2 years were separately 0.67 (95% CI: 0.54 - 0.84) and 0.78 (95% CI: 0.69 – 0.88) at the follow up of 3 and 5 years, compared to women without metformin usage. Similarly, when keeping the other covariates constant, model 2 illustrates that HRs for women who used statins for 2 years were separately 0.72 (95% CI: 0.58 - 0.91) and 0.90 (95% CI: 0.79 – 1.02) at the follow up of 3 and 5 years, compared to women without statins usage. These plots show a negative association of metformin (or statins) with breast cancer, and how the significance of estimates diminished if the patient survives long enough.

**Table S1 Primary analyses: Coefficients of metformin, statins and their interaction on the risk of breast cancer in Cox models (No. of patients with T2DM = 29,498; No. of patients diagnosed with breast cancer: 515).**

| **cumulative exposure in years** |  | **Crude model ^†^** | |  | **Model 1 ^‡^** | | | | |  | **Model 2** **^§^** | | | | |
| --- | --- | --- | --- | --- | --- | --- | --- | --- | --- | --- | --- | --- | --- | --- | --- |
|  |  |  |  |  | **without interaction ^¶^** | |  | **with interaction** | |  | **without interaction ^¶^** | |  | **with interaction** | |
|  |  | **HR (95% CI)** | **P** |  | **HR (95% CI)** | **P** |  | **HR (95% CI)** | **P** |  | **HR (95% CI)** | **P** |  | **HR (95% CI)** | **P** |
| metformin * statins |  | - | - |  | - | - |  | 0.97 [0.95, 0.99] | 0.001 |  | - | - |  | 0.97 [0.96, 0.99] | 0.001 |
|  |  |  |  |  |  |  |  |  |  |  |  |  |  |  |  |
| metformin |  | 0.41 [0.34, 0.50] | <0.001 |  | 0.46 [0.38, 0.55] | <0.001 |  | 0.47 [0.38, 0.56] | <0.001 |  | 0.45 [0.37, 0.54] | <0.001 |  | 0.45 [0.37, 0.55] | <0.001 |
| metformin * time basis_1 |  | 1.76 [1.44, 2.16] | <0.001 |  | 1.65 [1.34, 2.02] | <0.001 |  | 1.68 [1.37, 2.06] | <0.001 |  | 1.67 [1.36, 2.05] | <0.001 |  | 1.70 [1.38, 2.08] | <0.001 |
| metformin * time basis_2 |  | 2.12 [1.72, 2.62] | <0.001 |  | 1.92 [1.55, 2.38] | <0.001 |  | 2.04 [1.65, 2.53] | <0.001 |  | 1.97 [1.59, 2.44] | <0.001 |  | 2.09 [1.68, 2.59] | <0.001 |
| metformin * time basis_3 |  | 2.24 [1.84, 2.73] | <0.001 |  | 2.01 [1.65, 2.45] | <0.001 |  | 2.21 [1.79, 2.72] | <0.001 |  | 2.08 [1.70, 2.54] | <0.001 |  | 2.28 [1.85, 2.81] | <0.001 |
|  |  |  |  |  |  |  |  |  |  |  |  |  |  |  |  |
| statins |  | 0.45 [0.37, 0.54] | <0.001 |  | 0.51 [0.42, 0.62] | <0.001 |  | 0.52 [0.43, 0.63] | <0.001 |  | 0.47 [0.39, 0.58] | <0.001 |  | 0.48 [0.40, 0.59] | <0.001 |
| statins * time basis_1 |  | 1.70 [1.38, 2.08] | <0.001 |  | 1.57 [1.28, 1.93] | <0.001 |  | 1.60 [1.31, 1.97] | <0.001 |  | 1.62 [1.32, 1.99] | <0.001 |  | 1.65 [1.35, 2.03] | <0.001 |
| statins * time basis_2 |  | 2.07 [1.68, 2.56] | <0.001 |  | 1.87 [1.51, 2.31] | <0.001 |  | 1.97 [1.59, 2.43] | <0.001 |  | 1.96 [1.59, 2.42] | <0.001 |  | 2.06 [1.67, 2.55] | <0.001 |
| statins * time basis_3 |  | 2.22 [1.82, 2.71] | <0.001 |  | 1.98 [1.63, 2.42] | <0.001 |  | 2.16 [1.76, 2.65] | <0.001 |  | 2.10 [1.72, 2.56] | <0.001 |  | 2.28 [1.85, 2.80] | <0.001 |

**^†^** Crude model: include cumulative exposure to metformin and its interaction with time; **^‡^** Model 1: include cumulative exposure to metformin and statins, and their interaction with time, with and without the interaction term between metformin and statins; **^§^** Model 2: additionally adjust baseline information, i.e., age, bmi, smoking status, duration of diabetes, a history of cardiovascular diseases, a history of hypertension, calendar year, as well as updated values of HbA1c, LDL-C and eGFR, and cumulative exposure to sulfonylurea and insulin to Model 1. **^¶^** Without interaction refers to without including the interaction term between metformin and statins into the model.

Abbreviations: HR, hazard ratio; CI, confidence interval.

**Table S2 Sensitivity analyses: Coefficients of metformin, statins and their interaction on the risk of breast cancer in Cox model 2 with or without interaction term.**

| **cumulative exposure in years** | |  | **Model 2: without interaction ^†^** | | | | |  | **Model 2: with interaction** | | | | |
| --- | --- | --- | --- | --- | --- | --- | --- | --- | --- | --- | --- | --- | --- |
|  |  |  | **women aged ≥55 years old**  **^‡^** | |  | **women with duration of diabetes <2 years**  **^‡^** | |  | **women aged ≥55 years old**  **^‡^** | |  | **women with duration of diabetes <2 years**  **^‡^** | |
|  |  |  | **HR (95% CI)** | **P** |  | **HR (95% CI)** | **P** |  | **HR (95% CI)** | **P** |  | **HR (95% CI)** | **P** |
| metformin * statins | |  | - | - |  | - | - |  | 0.97 [0.95, 0.99] | 0.003 |  | 0.98 [0.95, 1.00] | 0.099 |
|  |  |  |  |  |  |  |  |  |  |  |  |  |  |
| metformin | |  | 0.44 [0.35, 0.54] | <0.001 |  | 0.41 [0.29, 0.59] | <0.001 |  | 0.44 [0.36, 0.55] | <0.001 |  | 0.41 [0.29, 0.59] | <0.001 |
| metformin * time basis_1 | |  | 1.72 [1.38, 2.15] | <0.001 |  | 1.93 [1.34, 2.80] | 0.001 |  | 1.76 [1.41, 2.19] | <0.001 |  | 1.95 [1.35, 2.82] | <0.001 |
| metformin * time basis_2 | |  | 2.02 [1.61, 2.53] | <0.001 |  | 2.19 [1.48, 3.24] | <0.001 |  | 2.14 [1.70, 2.69] | <0.001 |  | 2.29 [1.54, 3.41] | <0.001 |
| metformin * time basis_3 | |  | 2.07 [1.68, 2.57] | <0.001 |  | 2.23 [1.55, 3.21] | <0.001 |  | 2.27 [1.82, 2.83] | <0.001 |  | 2.42 [1.66, 3.52] | <0.001 |
|  |  |  |  |  |  |  |  |  |  |  |  |  |  |
| statins |  |  | 0.44 [0.36, 0.54] | <0.001 |  | 0.38 [0.26, 0.54] | <0.001 |  | 0.45 [0.36, 0.55] | <0.001 |  | 0.38 [0.26, 0.54] | <0.001 |
| statins * time basis_1 | |  | 1.72 [1.39, 2.14] | <0.001 |  | 1.94 [1.34, 2.79] | 0.001 |  | 1.76 [1.42, 2.19] | <0.001 |  | 1.98 [1.37, 2.85] | <0.001 |
| statins * time basis_2 | |  | 2.09 [1.68, 2.61] | <0.001 |  | 2.60 [1.76, 3.84] | <0.001 |  | 2.19 [1.75, 2.74] | <0.001 |  | 2.70 [1.83, 3.99] | <0.001 |
| statins * time basis_3 | |  | 2.25 [1.82, 2.77] | <0.001 |  | 2.77 [1.93, 3.98] | <0.001 |  | 2.42 [1.95, 3.00] | <0.001 |  | 2.95 [2.03, 4.27] | <0.001 |

**^†^** Without interaction refers to without including the interaction term between metformin and statins into the model. **^‡^** Regarding the patients with age ≥55 years old, the sample size was 24,336; of them, 457 women were diagnosed breast cancer. As for women with duration of diabetes <2 years, the sample size was 12,838; of them, 224 women got breast cancer diagnosis.

Abbreviations: HR, hazard ratio; CI, confidence interval.

**Table S3 multivariate Cox model for examining the effect of statins on the risk of breast cancer separately at 3-, 5-, 7- and 10-years follow up.**

| **Follow up** |  | **Crude model ^†^** | |  | **Model 1 ^‡^** | | | **Model 2 ^§^** | |
| --- | --- | --- | --- | --- | --- | --- | --- | --- | --- |
|  |  | **HR (95% CI)** | ***P*** |  | **HR (95% CI)** | ***P*** |  | **HR (95% CI)** | ***P*** |
| **3-year** |  |  |  |  |  |  |  |  |  |
| **without interaction with metformin ^¶^** |  | 0.85 [0.77, 0.92] | <0.001 |  | 0.88 [0.80, 0.96] | 0.005 |  | 0.85 [0.77, 0.93] | 0.001 |
| **with interaction with metformin** |  |  |  |  |  |  |  |  |  |
| 0 |  | - | - |  | 0.92 [0.84, 1.01] | 0.078 |  | 0.89 [0.81, 0.98] | 0.015 |
| 1 |  | - | - |  | 0.89 [0.82, 0.98] | 0.012 |  | 0.86 [0.79, 0.95] | 0.002 |
| 3 |  | - | - |  | 0.84 [0.77, 0.92] | <0.001 |  | 0.81 [0.74, 0.89] | <0.001 |
| 5 |  | - | - |  | 0.79 [0.71, 0.88] | <0.001 |  | 0.77 [0.69, 0.85] | <0.001 |
| **5-year** |  |  |  |  |  |  |  |  |  |
| **without interaction with metformin ^¶^** |  | 0.94 [0.89, 0.99] | 0.029 |  | 0.97 [0.91, 1.02] | 0.260 |  | 0.95 [0.89, 1.01] | 0.076 |
| **with interaction with metformin** |  |  |  |  |  |  |  |  |  |
| 0 |  | - | - |  | 1.04 [0.97, 1.12] | 0.244 |  | 1.02 [0.95, 1.10] | 0.611 |
| 1 |  | - | - |  | 1.01 [0.95, 1.08] | 0.703 |  | 0.99 [0.93, 1.05] | 0.755 |
| 3 |  | - | - |  | 0.95 [0.90, 1.01] | 0.091 |  | 0.93 [0.88, 0.99] | 0.021 |
| 5 |  | - | - |  | 0.90 [0.84, 0.96] | 0.002 |  | 0.88 [0.82, 0.95] | <0.001 |
| **7-year** |  |  |  |  |  |  |  |  |  |
| **without interaction with metformin ^¶^** |  | 0.98 [0.92, 1.03] | 0.406 |  | 1.00 [0.94, 1.06] | 0.954 |  | 0.98 [0.92, 1.04] | 0.558 |
| **with interaction with metformin** |  |  |  |  |  |  |  |  |  |
| 0 |  | - | - |  | 1.09 [1.01, 1.18] | 0.024 |  | 1.07 [0.99, 1.16] | 0.081 |
| 1 |  | - | - |  | 1.06 [0.99, 1.14] | 0.082 |  | 1.04 [0.97, 1.12] | 0.234 |
| 3 |  | - | - |  | 1.00 [0.95, 1.06] | 0.992 |  | 0.98 [0.93, 1.04] | 0.589 |
| 5 |  | - | - |  | 0.94 [0.88, 1.01] | 0.072 |  | 0.93 [0.87, 0.99] | 0.028 |
| **10-year** |  |  |  |  |  |  |  |  |  |
| **without interaction with metformin ^¶^** |  | 1.02 [0.93, 1.13] | 0.637 |  | 1.04 [0.93, 1.15] | 0.503 |  | 1.02 [0.92, 1.13] | 0.679 |
| **with interaction with metformin** |  |  |  |  |  |  |  |  |  |
| 0 |  | - | - |  | 1.16 [1.02, 1.30] | 0.019 |  | 1.14 [1.01, 1.28] | 0.038 |
| 1 |  | - | - |  | 1.12 [1.00, 1.26] | 0.045 |  | 1.10 [0.99, 1.23] | 0.084 |
| 3 |  | - | - |  | 1.06 [0.95, 1.17] | 0.288 |  | 1.04 [0.94, 1.15] | 0.431 |
| 5 |  | - | - |  | 1.00 [0.90, 1.10] | 0.929 |  | 0.98 [0.89, 1.09] | 0.741 |

**^†^** Crude model: include cumulative exposure to statins and its interaction with time; **^‡^** Model 1: include cumulative exposure to metformin and statins, and their interaction with time, with and without the interaction term between metformin and statins; **^§^** Model 2: additionally adjust for baseline information, i.e., age, BMI, smoking status, duration of diabetes, a history of cardiovascular diseases, a history of hypertension, calendar year, as well as updated values of HbA1c, LDL-C, and eGFR, and cumulative exposure to sulfonylurea and insulin to Model 1.  **^¶^** Without interaction refers to without including the interaction term between metformin and statins into the model.

Abbreviations: CI, confidence interval; HR, hazard ratio.

**Table S4 Sensitivity analyses with inverse probability weights: Coefficients of metformin, statins and their interaction on the risk of breast cancer in Cox model 2 with or without the interaction term** **^‡^**.

| **cumulative exposure in years** | |  | **Model 2: without interaction ^†^** | |  | **Model 2: with interaction** | |
| --- | --- | --- | --- | --- | --- | --- | --- |
|  |  |  | **HR (95% CI)** | **P** |  | **HR (95% CI)** | **P** |
| metformin * statins | |  | - | - |  | 0.97 [0.95, 0.99] | 0.008 |
|  |  |  |  |  |  |  |  |
| metformin | |  | 0.43 [0.35, 0.52] | <0.001 |  | 0.43 [0.35, 0.53] | <0.001 |
| metformin * time basis_1 | |  | 1.69 [1.37, 2.10] | <0.001 |  | 1.73 [1.39, 2.14] | <0.001 |
| metformin * time basis_2 | |  | 2.03 [1.63, 2.52] | <0.001 |  | 2.14 [1.71, 2.68] | <0.001 |
| metformin * time basis_3 | |  | 2.18 [1.77, 2.69] | <0.001 |  | 2.37 [1.90, 2.96] | <0.001 |
|  |  |  |  |  |  |  |  |
| statins |  |  | 0.51 [0.41, 0.62] | <0.001 |  | 0.51 [0.42, 0.63] | <0.001 |
| statins * time basis_1 | |  | 1.59 [1.28, 1.98] | <0.001 |  | 1.63 [1.31, 2.01] | <0.001 |
| statins * time basis_2 | |  | 1.85 [1.48, 2.30] | <0.001 |  | 1.94 [1.56, 2.43] | <0.001 |
| statins * time basis_3 | |  | 1.93 [1.57, 2.38] | <0.001 |  | 2.10 [1.69, 2.61] | <0.001 |

**^†^** Without interaction refers to without including the interaction term between metformin and statins into the model. **^‡^** the regression model included metformin and statins, with or without their interaction, when considering the estimated inverse probability weights at the same time.

Abbreviations: HR, hazard ratio; CI, confidence interval.

**Figure S1**

**
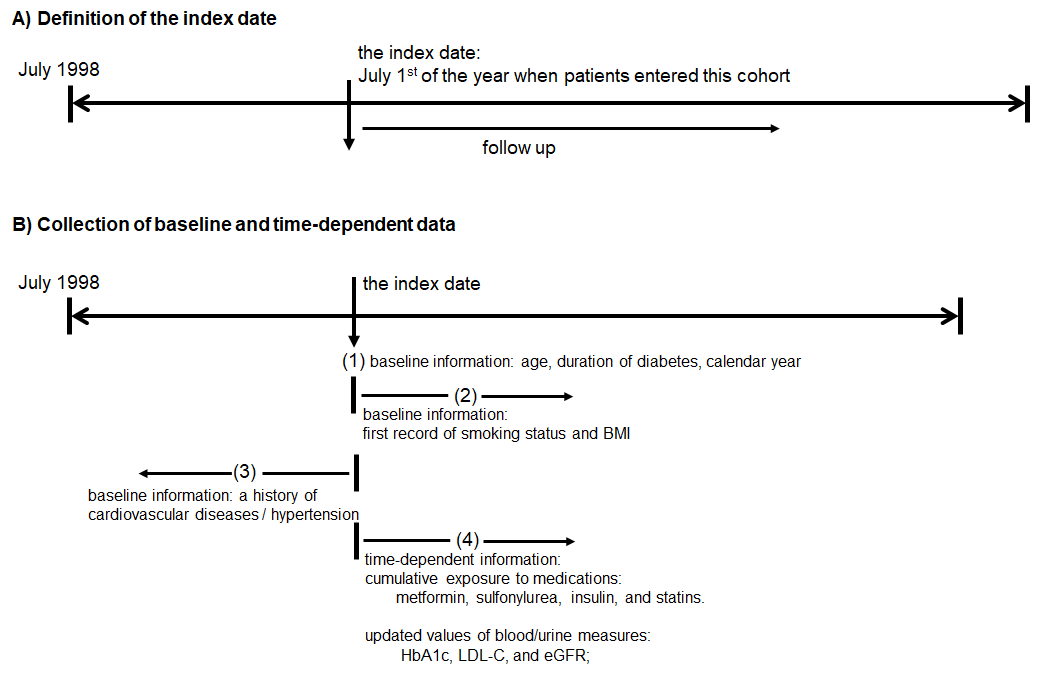
**

**Figure S2**

**
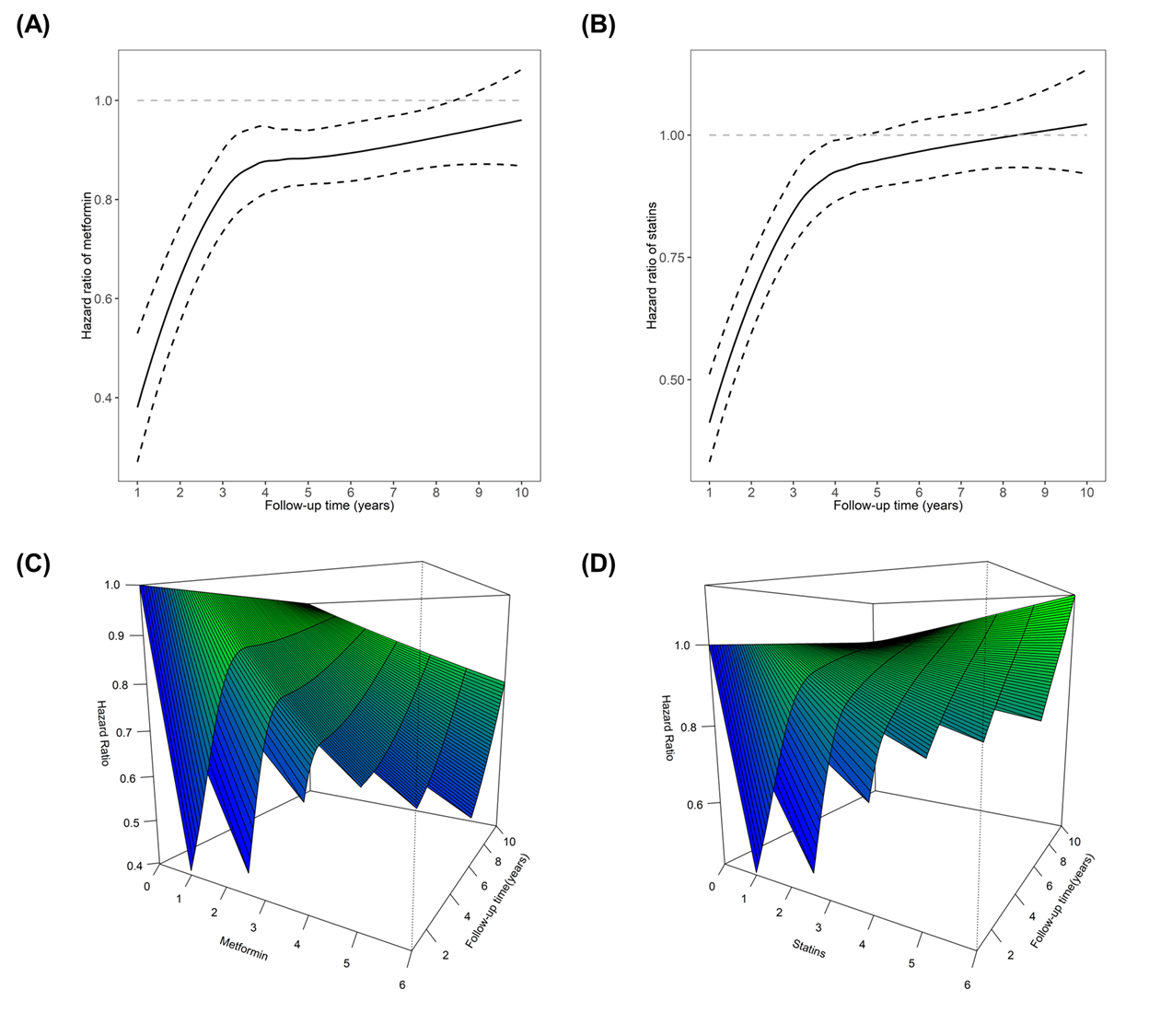
**
